# Retraction: Soil organic carbon and labile and recalcitrant carbon fractions attributed by contrasting tillage and cropping systems in old and recent alluvial soils of subtropical eastern India

**DOI:** 10.1371/journal.pone.0272536

**Published:** 2022-08-17

**Authors:** The PLOS ONE Editors

The *PLOS ONE* Editors retract this article [1] because it was identified as one of a series of submissions for which we have concerns about authorship, competing interests, and peer review. We regret that the issues were not addressed prior to the article’s publication.

RS, DS, AKS, SD, KB, PM, MJA, and RD did not agree with the retraction. SHS and PMB either did not respond directly or could not be reached.

## References

[1] R S., Sarkar D, Sinha AK, Danish S, Bhattacharya PM, Mukhopadhyay P, et al. (2021) Soil organic carbon and labile and recalcitrant carbon fractions attributed by contrasting tillage and cropping systems in old and recent alluvial soils of subtropical eastern India. PLoS ONE 16(12): e0259645. 10.1371/journal.pone.0259645 34914729 PMC8675705

